# Herbal medicine for the prevention of sarcopenia: A protocol for systematic review

**DOI:** 10.1097/MD.0000000000025474

**Published:** 2021-04-09

**Authors:** Jin-Ho Jeong, Ji Hye Hwang

**Affiliations:** a Jisung-Kyunghee Korean Medicine Clinic, Seoul; b Department of Acupuncture and Moxibustion Medicine, College of Korean Medicine, Gachon University, Seongnam, Republic of Korea.

**Keywords:** herbal medicine, muscle health, protocol, sarcopenia, systematic review

## Abstract

**Background:**

Sarcopenia is a syndrome characterized by a gradual and generalized loss of skeletal muscle mass and strength leading to physical disability, poor quality of life, and possible death. The disease impacts the steadily increasing aging population worldwide. In this systematic review protocol, we aim to investigate the effects and safety of herbal medicines on skeletal muscle health, specifically focusing on possible treatments for preventing sarcopenia.

**Methods:**

Nine electronic databases will be comprehensively searched from inception to the present date. Clinical studies reporting on the effects and safety of herbal medicines associated with skeletal muscle health and the prevention of sarcopenia will be included. The primary outcome will be determined by five categories: anti-inflammatory and antioxidant efficacy, muscle damage prevention, anti-fatigue effect, muscle-atrophy prevention, and muscle regeneration and differentiation. Two independent researchers will perform the research selection, data extraction, and quality assessment processes. The Cochrane risk-of-bias tool will be used to assess the methodological quality and design of the included studies. We will place no restrictions on publication status or language, and the results of the systematic review will be disseminated electronically and in print by publication in a peer-reviewed journal.

**Results:**

The present study will evaluate the effects and safety of herbal medicines for the prevention of sarcopenia.

**Conclusion:**

Our findings will provide guidance on the use of herbal medicines to prevent sarcopenia. This knowledge is valuable for elderly people, clinicians treating patients with sarcopenia, policy makers, and researchers.

**Registration number:**

Reviewregistry1113 (URL: https://www.researchregistry.com/browse-the-registry#registryofsystematicreviewsmeta-analyses/registryofsystematicreviewsmeta-analysesdetails/604a1e5ef176d2001b557750/).

## 1 Introduction

Population aging is occurring worldwide in all regions and countries, despite different levels of development.^[1]^ Moreover, the proportion of the population over 60 is increasing yearly and is expected to double by 2050.^[2]^ As one of the most important trends of the 21st century, population aging has a significant and far-reaching impact on all aspects of society.^[1]^ The gradual loss of skeletal muscle mass, muscle strength, and/or muscle function affects older adults, and is a particular concern for obese older adults. These types of age-related changes may be due to sarcopenia since it causes a loss of muscle protein mass and function with increasing age,^[3–5]^ and is a key component in the development of frailty.^[6]^ Sarcopenia is also a syndrome characterized by a gradual and generalized loss of skeletal muscle mass and strength placing older individuals at risk for adverse consequences such as disability, poor quality of life, and possible death.^[7]^ Factors implicated in the etiology of sarcopenia include decreased physical activity, malnutrition, increased cytokine activity, oxidative stress, and abnormalities in growth hormone and sex steroid axes.^[8]^ The approaches most commonly applied to slow the progression of sarcopenia have been based on a combination of regular exercise programs and proper nutrition related to the use of dietary supplements.^[9]^

Despite a long history of traditional medicine use and the major benefits of natural remedies, few studies have been conducted on the relationship between herbal medicines and age-related pathologies of skeletal muscle.^[10]^ In 2016, a systematic review of botanicals related to sarcopenia prevention including in vivo, in vitro, and human studies was conducted only on English publications found in the PubMed and Scopus databases.^[11]^

Our systematic review aims to investigate the effects and safety of herbal medicines on skeletal muscle health and prevention of sarcopenia in published human studies, regardless of language. Our focus will be on possible treatments using methods such as traditional Chinese medicine (TCM) and Korean medicine (KM).

## 2 Methods

### 2.1 Study registration

This systematic review will be conducted in accordance with the Preferred Reporting Items for Systematic Reviews and Meta-Analyses protocols (PRISMA-P).^[12]^ The protocol was registered in the Research Registry (registration number: reviewregistry1113) (https://www.researchregistry.com/browse-the-registryofsystematicreviewsmeta-analyses/).

### 2.2 Ethics and dissemination

The study will review published literature, and because no patient recruitment or personal data collection will occur, no ethical approval is required. The results of this systematic review will be disseminated by publication in a peer-reviewed journal, or presentation at a relevant conference.

### 2.3 Eligibility criteria

#### 2.3.1 Types of participants

Human studies (including postmenopausal women, athletes, and others) focusing on possible treatments related to skeletal muscle health for prevention of sarcopenia will be included. As described in a previous review study,^[11]^ therapeutic effects will be analyzed in detail based on five categories: anti-inflammatory and antioxidant efficacy, muscle damage prevention, anti-fatigue effect, muscle atrophy prevention, and muscle regeneration and differentiation.

#### 2.3.2 Types of interventions and controls

The reviewed studies will include patients treated with herbal medicine alone, and also those treated concurrently with herbal medicine and other therapies. Concurrent treatments will be considered acceptable only if herbal medicine is applied to the intervention group, and other treatments are provided equally to both the intervention and control groups. Studies evaluating any form of herbal medicine, such as decoctions, tablets, capsules, extracts, powders, or pills will be eligible for inclusion.

For control groups, we will consider placebo or sham, no interventions, and any type of control intervention compared with herbal medicine. Studies that do not list the compositions of the herbal medicines will be excluded, except for patented drugs.

#### 2.3.3 Types of studies

Original clinical studies, including randomized controlled clinical trials, non-randomized controlled clinical trials, and before-after studies to assess the beneficial effects and safety of herbal medicine for the prevention of sarcopenia will be included.

#### 2.3.4 Outcomes and prioritization

The primary outcomes will be analyzed in detail according to the following five categories:

1. Anti-inflammatory and antioxidant properties: levels of inflammatory cytokines TNF-α, sTNF-RII, IL-6, IL-1ra, IL-8, etc, and antioxidant enzyme levels.
2. Prevention of muscle damage: delayed-onset muscle soreness (DOMS), acceleration in recovery of muscle strength following intense exercise, etc.
3. Anti-fatigue effect: serum lipid profiles, blood urea nitrogen (BUN), etc.
4. Prevention of muscle atrophy: skeletal muscle mass, muscle atrophy after intense exercise, muscle strength, risk of mobility impairments, etc.
5. Muscle regeneration and differentiation effects: skeletal muscle mass, hand grip strength, ratio of plasma follistatin/myostatin, improvement of several physical conditions, etc.

### 2.4 Data sources and search strategy

**Figure 1:**
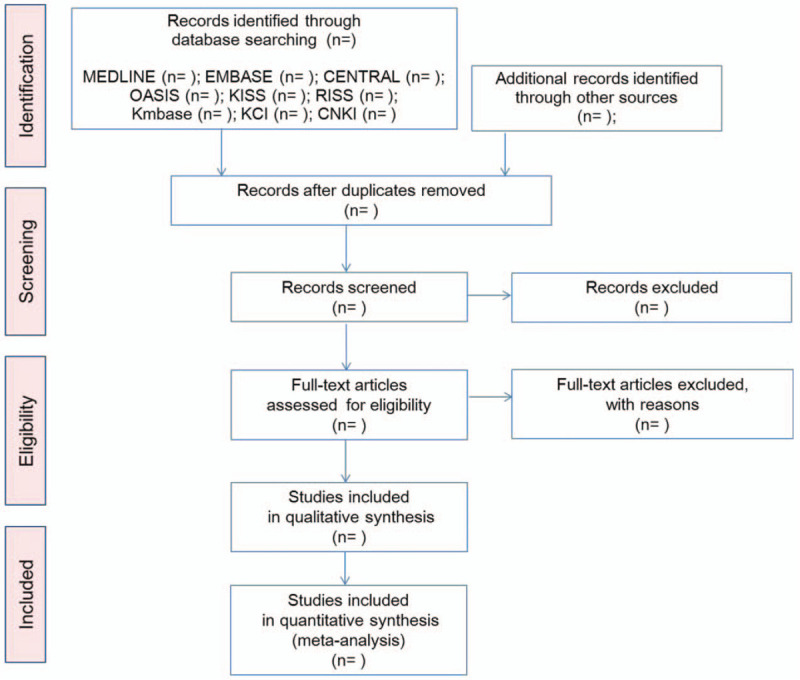
A Preferred Reporting Items for Systematic Reviews and Meta-Analyses protocols (PRISMA-P) flow diagram of the literature screening and selection processes. CENTRAL = Cochrane Central Register of Controlled Trials, CNKI = China National Knowledge Infrastructure, KCI = Korea Citation Index, KISS = Korean Studies Information Service System, KMbase = Korean Medical Database, OASIS = Oriental Medicine Advanced Searching Integrated System.

Databases and search terms will be determined after discussions between all authors, and before the literature search is executed. Two independent researchers will perform the electronic literature searches, study selection, data extraction, and quality assessment. The following electronic databases will be searched for studies from inception to the present date: Medline via PubMed, EMBASE via Elsevier, the Cochrane Central Register of Controlled Trials, Oriental Medicine Advanced Searching Integrated System (OASIS), Korean Studies Information Service System (KISS), Research Information Service System (RISS), Korean Medical Database (KMbase), Korea Citation Index (KCI), and China National Knowledge Infrastructure (CNKI) (Fig. 1). Any disagreements between the two researchers will be resolved by consensus. No restrictions will be placed on publication status or language.

### 2.5 Data extraction

All articles will be reviewed to evaluate inclusion eligibility, and in the case of uncertainties, the authors will be contacted for further information. After study selection, we will extract the following data: author, year of publication, study design, participants (age, sex), diseases or disorders, control intervention, outcome measures, main results, and adverse events.

Two reviewers will conduct data extraction using a recognized data extraction form agreed upon by all reviewers that will include author names, ages, country, year of publication, characteristics of participants, interventions, randomization methods, blinding, control treatments, main outcomes, and adverse events. The reviewers will perform a quality assessment using the predefined data extraction form. Any disagreements between the two researchers will be resolved by consensus.

### 2.6 Data synthesis and analysis

Differences between intervention and control groups will be assessed. Mean differences (MDs) with 95% confidence intervals (CIs) will be used to measure treatment effects for continuous data. We will convert other forms of data into MDs. For outcome variables on different scales, we will use standard MDs with 95% confidence intervals. For dichotomous data, we will present treatment effects as relative risks (RRs) with 95% CIs; other binary data will be converted into RR values.

All statistical analyses will be conducted using the Cochrane Collaboration software program Review Manager (version 5.3; The Nordic Cochrane Center, the Cochrane Collaboration, 2014, Copenhagen, Denmark) for Microsoft Windows (http://www.microsoft.com). We will contact the corresponding authors of studies with missing information to acquire and verify the data whenever possible. When appropriate, we will pool data across studies to conduct a meta-analysis using fixed or random effects. We will use the GRADEpro software from Cochrane Systematic Reviews to create a Summary of Findings table.

The original source or published trial reports for the data will be reviewed in cases where individual patient data are initially unavailable.

### 2.7 Assessment of risk-of-bias in individual studies

We will use the Cochrane Collaboration risk-of-bias (RoB) tool to assess the RoB of the included randomized controlled clinical trials. Domains including random sequence generation, allocation concealment, blinding of participants, personnel, outcome assessors, completeness of data outcome, selective reporting, and other biases will be assessed as “low risk,” “unclear risk,” or “high risk.” We will assess other bias items as the statistical baseline imbalance severity between the treatment and control groups, including the mean age, sex, disease period, and/or disease severity of the participant. The RoB in nonrandomized studies determined by using the interventions tool will be used to assess the RoB of included nonrandomized controlled clinical trials. The quality assessment tool for before-after (pre-post) studies with no control group, proposed by the National Heart, Lung, and Blood Institute, will be used to assess the RoB of included before-after studies. Two researchers will independently assess the methodological quality of the included studies, and any discrepancies will be resolved by consensus.

### 2.8 Analysis of subgroups or subsets

Subgroup analyses will be performed if considerable heterogeneity is identified (defined by the results of heterogeneity tests indicating *P* < .1, via χ^2^ tests and Higgins *I*^2^ ≥ 50%).

## 3 Discussion

The term sarcopenia (Greek, sarx for “flesh” and penia for “loss”) describes the phenomenon of decreasing muscle mass and function with aging.^[13]^ Sarcopenia is mainly considered an inevitable part of aging, and is a major determinant of fall risk and impaired ability to perform daily activities that often leads to disability, loss of independence, and possible death. Despite its clinical significance, sarcopenia is often unrecognized and poorly managed in routine clinical practice, due in part to the lack of available diagnostic tests and consistent diagnostic criteria. The management of sarcopenia focuses primarily on physical therapy for muscle strengthening and gait training, and currently no approved pharmacological agents for its treatment are available.^[14]^

In this systematic review, we will investigate clinical studies for the effects and safety of herbal medicines associated with skeletal muscle health and prevention of sarcopenia by assessing five categories: anti-inflammatory and antioxidant efficacy, muscle damage prevention, anti-fatigue effect, muscle atrophy prevention, and muscle regeneration and differentiation. We expect that our findings will be a useful resource to suggest optimized clinical treatment strategies for health policy makers, clinical practitioners, patients, and researchers. Healthcare-based improvements in the quality of life of the elderly will be beneficial as the population ages, and our expectation is that patients with sarcopenia will receive appropriate herbal medicines from physicians according to clinical evidence.

## Author contributions

JHH conceived the study and developed the criteria, and JHH and JHJ searched the literature and analyzed the data. JHH wrote the protocol, and JHH and JHJ revised the manuscript. All authors have read and approved the final manuscript.

**Conceptualization:** jihye hwang.

**Data curation:** jihye hwang.

**Formal analysis:** jihye hwang.

**Investigation:** jihye hwang, Jin-Ho Jeong.

**Methodology:** jihye hwang.

**Project administration:** jihye hwang.

**Visualization:** jihye hwang.

**Writing – original draft:** jihye hwang.

**Writing – review & editing:** jihye hwang, Jin-Ho Jeong.
